# Habilitation and assistive devices for persons with disabilities: a new approach to respect for human rights and inclusion

**DOI:** 10.3389/fresc.2026.1783746

**Published:** 2026-08-31

**Authors:** Giampiero Griffo

**Affiliations:** 1 Centre for Governmentality and Disability Studies ‘Robert Castel’ - CeRC - University Suor Orsola Benincasa Naples Italy, Disabled People's International-DPI, Strabourg, France; 2 CeRC, Suor Orsola Benincasa University of Naples, Naples, Italy

**Keywords:** assistive devices, UNCRPD, post-human, habilitation, functional profile, assessment, human rights

## Abstract

In recent years, the development of assistive technologies has profoundly changed the idea of what it means to be human and what humans are capable of, in particular for persons with disabilities; the United Nations Convention on the Rights of Persons with Disabilities (UNCRPD) highlights that disability is a social construction. New concepts have been developed, such as empowerment, enabling, inclusion, habilitation, and human rights, that have changed the meaning of making tools available to encourage full participation, hoping to solve challenges of today, such as barriers, obstacles, and discrimination. The development of assistive devices has reconfigured the limits of the human body for all persons and has made it clear that persons with functional limitations can overcome the body's hereditary materiality limits, activating enabling and resilient factors that configure a different normality of “doing”. The post-human perspectives question traditional rehabilitation forms, which are no longer centered on recovering a lost or limited function according to a predefined model of “normality” and health (ableism), but reformulating the idea of how a person can function. It is from the analysis of the human being’s functioning profile that new forms of protection for persons with disabilities and their human rights can be explored, such as the customized participatory projects envisaged by the Italian welfare reform legislation. In this context, Article 26 of the UNCRPD proposes a new approach to support the human rights and inclusion of persons with disabilities. Assistive devices are no longer a tool linked to the health sector, but rather allow us to expand the ability to participate and broaden functional capabilities and should therefore be provided in a social context, as useful tools to achieve full citizenship.

## Habilitation and assistive devices for persons with disabilities: a new approach on human rights respect and inclusion

In recent years, the development of digital technologies has profoundly changed the idea of how we define human beings. Most of these technologies extend the natural capabilities of the human body, expanding their field of action and activities. This has happened in the field of communication and information (internet and mobile phones), document production and their archiving (computers), activities carried out remotely with communication platforms, and a variety of other activities such as telemedicine, technical and professional consultancy, remote controls, the definition of assistive software, and app development. This revolution has been described as the creation of neuronal extensions of the human body and new forms of intelligence^1^, as well as a vast field of analysis that is defined as posthuman^2^, which includes philosophical reflections and social aspects and begins to prefigure profound changes in the way future societies develop.

### The posthuman and persons with disabilities

These new perspectives have important consequences for persons with disabilities, both in the field of access to these aids and in the cultural and political interpretations of how to identify and assign them. At the same time, Article 26 of the United Nations Convention on the Rights of Persons with Disabilities (UNCRPD) reformulates the concept of assessment and the possibility of empowering persons with disabilities through appropriate, customized participatory support.

The current definition of aids is linked to the medical-rehabilitation field. They arise from evaluations based on body interventions: the current distinction identifies prostheses (an artificial device designed to replace a missing part of the body—a limb, an organ or a tissue—or to integrate a damaged part) and orthoses (a medical device, a brace, orthopedic equipment, or similar used in orthopedics, orthodontics, or traumatology in the treatment of certain pathologies). In practice, the two types of aid are centered on the functioning of the human body in order to recover its compromised functions. Over time, new materials and new designs have been used, improving performance, sometimes surprisingly^3^.

The United Nations Convention on the Rights of Persons with Disabilities (2006) then transformed the definition of disability as a social construction. In Article 1, paragraph 2, persons with disabilities are defined as “those who have long-lasting physical, mental, intellectual or sensory impairments which in interaction with barriers of a different nature can hinder their full and effective participation in society on an equal basis with others”^4^. From this definition, new concepts have been developed on how to guarantee the full participation of these persons in society, no longer centered on the individual, but rather on interaction with the environment and the living context. The recognition that persons with disabilities share the same human rights on an equal basis as other citizens (Articles 1 and 5 of the UNCRPD) has highlighted how persons with disabilities are impoverished by negative social stigmas^5^ and by barriers, obstacles, and discrimination produced by society. New concepts have emerged, such as those of empowerment^6^ and inclusion, that permeate the entire UNCRPD, which underlines that the rights of these people must be included in all general policies that concern the entire population. Another important concept is that of habilitation. The Convention separates Article 26 on rehabilitation and habilitation from Article 25 on health. It is a cultural and technical revolution based on the idea that rehabilitation and habilitation are not limited to the healthcare sector and that disability is a social relationship, not a subjective personal condition, and therefore needs to be addressed by skills that touch on all relevant areas. The treatment objective to which they are subjected is to guarantee the highest possible level of health, but in a context in which health is not the absence of diseases or functional limitations, but the person's well-being^7^. Rather than dealing only with reconstructing a condition of presumed normality (ableism)^8^, people must be guaranteed the fundamental rights and freedoms enjoyment: they are rehabilitated if the functionality of the body that is compromised and/or lost is recovered; being habilitated is when new abilities are developed starting also from the functional limitation condition and from the set of characteristics that allow a person to function in a certain way and on which adequate skills and appropriate supports can be developed.

This approach transforms the idea of rehabilitative and social care: the UNCRPD recognizes “the equal right of all people with disabilities to live in the community, in equal conditions of choice compared to other members”; for this purpose, the 192 signatory states of the Convention (Article 19) “shall take effective and appropriate measures in order to facilitate the full enjoyment (…) of this right and full inclusion and participation within the community”, including the right “to choose their place of residence and where and with whom to live, on the basis of equality with others”, not being “obliged to live in a particular place”, “have access to a range of home, residential or community support services, including personal assistance necessary to support life and inclusion within the community and to prevent isolation or segregation outside the community”, and ensuring “community services and facilities for the whole population are available on an equal basis to persons with disabilities and are responsive to their needs”.

The application of the UNCRPD entails a profound transformation of current protection-based welfare to inclusion-based welfare; it was widely highlighted during the pandemic crisis that people with disabilities and their families have suffered a disproportionate burden of problems, as stated by the European Commissioner for Equity Helena Dalli^9^, and have been essentially unprotected^10^.

Article 26 reinforces this perspective and underlines the importance of self-determination for persons with disabilities^11^. The article states the following: “States Parties shall organize, strengthen and develop comprehensive services and programs for habilitation and rehabilitation, in particular in the fields of health, employment, education and social services (…) promote the development of initial and ongoing training for professionals and staff working in habilitation and rehabilitation services (…) promote the offer, knowledge and the use of support technologies and tools, designed for persons with disabilities, which facilitate their habilitation and rehabilitation”.

Once the limit of rehabilitation capabilities has been reached, the approach becomes an enabling approach to allow people to achieve levels of autonomy, self-determination, independence and inter-independence. Non-medical figures are key contributors to this objective because the habilitation process must involve experts in a wide range of areas, such as rights, education, employment, independent living, sport, etc.

Also in this case, for all persons with disabilities, a radical card transformation is taking place: from traditional institutionalization we move on to social systems of support for maintenance in living and family places, to family-based forms of reception, to social inclusion policies and services, and to personalized projects and support^12^. The direct participation of persons with disabilities or those who represent them is essential. It is a substantial change in the concept of taking charge: the person with disabilities chooses, like all citizens, the supports that favor their life plan. In the assessment of persons with disabilities, it is necessary to use the International Classification of Functioning, Disability and Health (ICF)^13^ in connection with the UNCRPD: the ICF is a nosographic instrument to evaluate the presence of persons with disabilities in a specific population, but it does not consider barriers and discrimination as a human rights violation, as the UNCRPD stresses^14^. Furthermore, the ICF is based on an ableist approach, starting from a comparison of the functioning of a person with disabilities with a body without functional limitations; the personal factors in the ICF are only mentioned but not developed. The habilitation process examines the functional profile in order to understand how, through appropriate support and training, they can carry out activities of their choice. This requires the direct participation of the persons concerned in the analysis and decision-making process. The habilitation therefore analyses how the person could carry out certain activities through personal forms of empowerment or through specific aids of various kinds (instrumental, technological, and human).

At the same time, Article 20 of the UNCRPD recognizes personal mobility as a human right of persons with disabilities, which, considering UNCRPD Articles 9 (accessibility) and 5 (equality and non-discrimination), requires profound changes to the administrative recognition procedures of these aids. Naturally, it is necessary for beneficiaries to be informed free of charge about the market evolution, with free information services on all new solutions, in order to choose the most appropriate products for the single person^15^.

The development of new technologies has highlighted the need to guarantee technical regulations that do not produce other barriers^16^. These new technologies have spread across the world, enhancing human capabilities regardless of the body's capabilities, starting the process of a post-human world that involves the entire human race.

The WHO and UNICEF definitions of assistive devices are very broad: “Assistive technology is an umbrella term for assistive products and their related systems and services. Assistive technology is of fundamental importance for persons with permanent or temporary functional difficulties as it improves their functional ability, and enables and enhances their participation and inclusion in all life domains. Assistive products may be physical products such as wheelchairs, spectacles, hearing aids, prostheses, walking devices or continence pads; or they may be digital, occurring in the form of software and apps that support interpersonal communication, access to information, daily time management, rehabilitation, education and training etc. They may also be adaptations to the physical environment, for example portable ramps or grab-rails. Definitions of assistive technology and assistive products differ depending on their purpose and scope”^17^.

The assistive devices concept has completely revolutionized the meaning of aids applied to persons with disabilities. This is because this new cultural and technical approach has reconfigured human beings' limits for all and has made it clear that persons with functional limitations can overcome the hereditary materiality body limits, activating enabling and resilient factors that configure a different normality of “doing” and of being.

The post-human perspectives question traditional rehabilitation forms: a person is no longer limited to recovering a lost or limited function according to a predefined model of “normality” and health, but the idea of how a person can function is reformulated, even considering body functional limitations, taking into account all its characteristics and appropriate supports.

The concept of habilitation introduced by Article 26 of the UNCRPD makes the meaning of Article 3, point d, of the UNCRPD clearer: “respect for difference and acceptance of persons with disabilities as part of human diversity and humanity itself ”. We can no longer be treated as people in whom only the psychophysical condition is highlighted, impoverishing the person who is reduced to that sole body or mind characteristics. Examples in human history allow us to understand this revolution: John Nash junior won a Nobel Prize in economics with his game theory despite living with schizophrenia (1994)^18^; Henri de Toulouse Lautrec was a great painter living with a congenital malformation^19^; Ludwig van Beethoven was a great musician who became deaf, but continued to compose^20^.

The change must therefore be understood as not reducing the person to a medical diagnosis. Medicine has also started to reflect on diagnoses, particularly in the United States, where precision medicine and precision psychiatry were born^21^. Persons with disabilities who have the same diagnoses may experience different levels of functioning, and this approach shifts attention to describing how people function to understand how that person has specificities that require personalized treatment.

It is precisely from the analysis of the human being's functioning profile that new forms of human rights protection for all persons with disabilities can be explored. To understand what we are talking about with habilitation, we should consider cosmologist Stephen Hawking as an example. Affected since 1963 by arterial spin labeling (ASL), he gradually lost his autonomy and ability to articulate words. By evaluating his functioning profile and abilities, his management plan involved personal assistance 24 h a day, moving with an electric wheelchair, and communicating through a computer that read the movement of the eyelids and eyes and translated this with speech synthesis software. Evaluating the elements of his way of functioning meant he could continue to do his job with the appropriate supports.

This profound revolution in the field of aid enables us understand how this new approach also changes the meaning of the provision of these products to persons with disabilities. Most of them no longer have to do with the health area, but with the areas of participation, independent living, and full citizenship. The 2022 WHO report, quantifying the current need for assistive devices at 2.5 billion people and predicting that it will reach 3.5 billion people in 2050^22^, says it explicitly: “access to appropriate, quality assistive technology can mean the difference between enabling or denying education for a child, participation in the workforce for an adult, or the opportunity to maintain independence and age with dignity for an older person”^23^.

We should start discussing whether the provision of assistive devices is entrusted to the field of habilitative activities, starting from the idea of intervening in the field of full participation and full citizenship, transferring resources from a health competence basis to one of social competence.

The new perspective related to habilitation and assistive devices supports the transition from hospital-based care, centered on the individual health condition, toward home- and community-centered care pathways, based on self-determination and interaction with the lives of persons with disabilities, respecting their human rights.

## Data Availability

The original contributions presented in the study are included in the article/Supplementary Material, further inquiries can be directed to the corresponding author.

## Footnotes

^1^See publications by Derrik de Kerckove, 1997, 2001, 2008, 2010 (9–12), and Buffardi, 2011 (5).

^2^The bibliography on this topic has expanded greatly; I will only cite some fundamental texts: Marchesini R. 2002 (13), with an extensive bibliography of the initial discussions; Adams C., Thompson T.L., 2016 (1); Zalloua Z., 2021 (18).

^3^Oscar Pistorius ran in the London Olympics in 2012 with prosthetics (“cheetahs”, a name inspired by cheetahs) on both legs and which used very expensive materials commonly used in aerospace engineering. These prostheses have raised controversy as to whether the South African runner was improperly advantaged by the use of these prostheses.

^4^See https://www.ohchr.org/en/instruments-mechanisms/instruments/convention-rights-persons-disabilities.

^5^Griffo G., 2014 (8).

^6^Empowerment is an English word that has two meanings: one linked to the person, of strengthening skills, competences, and motivations to be the protagonist of one's own life; the second is social and concerns acquiring power, participating in the life of one's community, and, therefore, contributing to ensuring that the rights of people with disabilities are respected. Persons with disabilities need both to strengthen their individual capacities and to gain greater power to decide how society includes them, through organizations of people with disabilities and their families.

^7^This is the WHO definition; see https://www.who.int/activities/promoting-well-being.

^8^Ableism is a cultural paradigm similar to racism and sexism, and it discriminates against people with disabilities. This concept assumes that all people have an able-bodied or “normal” body, that is, one that fits within the social and cultural conventions accepted by the community. The word "ableism" indicates a world designed and built without taking into consideration people who may have physical, mental, or other impediments. From this it follows that people who do not fit into the concept of "normality" are excluded from society, ghettoized, and discriminated against because they do not respond to the ordinary vision of things because they are "unskilled". See a complete bibliography on ableism at https://ableism.wordpress.com/ableism-bibliography/. Bellacicco et al. 2022 (3).

^9^See https://ec.europa.eu/commission/commissioners/2019-2024/dalli/announcements/speech-commissioner-dalli-impact-coronavirus-outbreak-persons-disabilities_en.

^10^See Borgia and Griffo, 2021 (4).

^11^The European Disability Forum and the European Union of Medical Specialists in Rehabilitation have collaborated on Article 26 based on a common document, see https://www.edf-feph.org/content/uploads/2025/04/EDF-Position-Paper-on-Health-Rehabilitation-and-Habilitation-of-Persons-with-Disabilities.pdf.

^12^In Italy, the enabling law on disability (n°227, 22 December 2021) goes in this direction.

^13^See https://www.who.int/standards/classifications/international-classification-of-functioning-disability-and-health#:∼:text=ICF%20is%20the%20WHO%20framework,and%20measure%20health%20and%20disability.

^14^Alves I. et al., 2010 (2).

^15^A good Italian example is the GLIC (Italian network of advised Centers of tehnological aids for persons with disabilities), https://www.centriausili.it/en/.

^16^See the international regulations which, through various technical instruments adopted at an international and national level, have defined how we guarantee accessibility to software, apps, websites, digital documents, etc.

^17^WHO and UNICEF (2022), page 5 (17).

^18^Nasar S., 2002 (15).

^19^Ryan M., 1991 (16).

^20^Caeyers J., 2020 (6).

^21^National Research Council, 2011 (14) and Fernandes et al., 2017 (7).

^22^The WHO estimates that there are 1.3 billion people with disabilities worldwide. If 2.5 billion people now need assistive devices, this means that these devices are intended for the entire population and not just for people with disabilities. This consideration leads to a shift in the use of assistive devices from healthcare tools used by people with functional limitations to ordinary aids useful to all of humanity. This helps to reduce the negative stigma that affects people with disabilities.

^23^WHO and UNICEF, 2022, page 7 (17).
